# Social representation of violence against women in psychology students at a university in the Colombian Caribbean

**DOI:** 10.1192/j.eurpsy.2023.1071

**Published:** 2023-07-19

**Authors:** Z. J. Miranda- Sánchez, D. I. Sánchez- Pabón, K. M. Múnera-Luque

**Affiliations:** Psychology, Universidad Cooperativa de Colombia, Santa Marta, Colombia

## Abstract

**Introduction:**

Violence against women is a violation of human rights and is part of one of the sustainable development goals. Thus, it is very important to be able to guarantee healthcare spaces from a differential approach, in which they will be developed that promote equality and will help to prevent violence. Therefore, it is necessary to analyse the social representation that future professionals will have in health, and that can affect the approach given to this phenomenon.

**Objectives:**

To analyse the social representations of violence against women in psychology students at a university in the Colombian Caribbean.

**Methods:**

The study was qualitative, exploratory and for convenience, with the application by web platform. The sample consisted of 110 psychology students from a university in the Colombian Caribbean, aged between 18 and 32 years (M=21; SD=3). The technique of free association of words and the application of semi-structured interviews were produced to identify the central and peripheral nucleus of social representation. For data analysis, the Atlas.ti version 22 software was obtained.

**Results:**

It was found that the social representation of violence against women, in its strongest association, deals with the types of physical violence, highlighting among these physical beatings, rapes, assaults and femicides. Likewise, the effects that this phenomenon generates on the mental health of the victims and its relationship with stereotypes about gender roles, in turn, the presence of problems in the judicial system, which end up causing many cases to go unpunished or re-victimize women.

**Conclusions:**

Violence against women constitutes a health problem, having professionals in this area who can understand the psychological impact, generates advantages in the development of strategies aimed at guaranteeing better care, which contributes not only to prevent this phenomenon but also to avoiding revictimization from mental health services.

**Disclosure of Interest:**

None Declared

